# Primary care effectiveness, multimorbidity, and hospital use in public and private health-care systems in Brazil: a cross-sectional analysis of data from the 2019–2021 Brazilian longitudinal study of aging

**DOI:** 10.1016/j.lana.2026.101680

**Published:** 2026-10-17

**Authors:** Giulia Muniz de Oliveira, Bruno Pereira Nunes, Kenneth Lam, Maria Fernanda Lima-Costa, Juliana Lustosa Torres, James Macinko, Bruno de Souza Moreira, Thiago Junqueira Avelino-Silva, Virgílio Augusto Deodato Gonçalves, Sileno de Queiroz Fortes-Filho, Thiago Figueiredo de Castro, Sandro Rogério Rodrigues Batista, Eduardo Ferriolli, Márlon Juliano Romero Aliberti

**Affiliations:** a Laboratorio de Investigacao Medica em Envelhecimento (LIM-66), Servico de Geriatria, Hospital das Clinicas HCFMUSP, Faculdade de Medicina, Universidade de Sao Paulo, Sao Paulo, Brazil; b Department of Epidemiology and Health Information, Hospital Israelita Albert Einstein, São Paulo, Brazil; c Department of Health and Kinesiology, University of Illinois Urbana-Champaign, Urbana, IL, USA; d Division of Geriatric Medicine, Department of Medicine, University of Colorado Anschutz Medical Campus, Aurora, CO, USA; e Center for Studies in Public Health and Aging, Universidade Federal de Minas Gerais and Fundação Oswaldo Cruz, Belo Horizonte, Brazil; f Departamento de Medicina Preventiva e Social, Universidade Federal de Minas Gerais, Belo Horizonte, Minas Gerais, Brazil; g Department of Health Policy and Management, Fielding School of Public Health, University of California, Los Angeles, Los Angeles, CA, USA; h Division of Geriatrics, Department of Medicine, University of California, San Francisco, San Francisco, CA, USA; i Research Institute, Hospital Sirio-Libanes, Sao Paulo, Brazil; j School of Health Sciences, Amazonas State University (UEA), Amazonas, Brazil; k Faculty of Medicine, University of Brasilia, Brasília, DF, Brazil; l Faculty of Medicine, Federal University of Goiás, Goiânia, GO, Brazil

**Keywords:** Multimorbidity, Access to primary care, Continuity of care, Hospitalization, Aging

## Abstract

**Background:**

Whether patient-reported experiences in primary care modify the association between multimorbidity and emergency department (ED) visits and hospitalizations remains unclear. We examined these associations across public and private health system sectors among Brazilians aged ≥50 years.

**Methods:**

This cross-sectional study used nationally representative data from the 2019–2021 Brazilian Longitudinal Study of Aging. Multimorbidity was defined as the presence of ≥2 of 13 chronic conditions. A 12-item patient-reported primary care effectiveness index captured access, continuity, communication, and coordination with participants’ usual source of healthcare. Primary outcomes were ED visits and hospitalizations in the past 12 months. Poisson regression models estimated associations with the outcomes, and assessed interactions among multimorbidity, primary care effectiveness, and health system sectors.

**Findings:**

Among 9058 participants (mean age = 66.3 ± 9.9 years; females = 55.3%; White race/ethnicity = 46.4%), 38.0% had multimorbidity. Overall, 12.5% reported an ED visit and 6.0% ≥ 1 hospitalization in the past 12 months. Multimorbidity was associated with approximately twice the prevalence of ED visits and three times the prevalence of hospitalizations. Primary care effectiveness modified these associations across health system sectors (three-way interaction p = 0.04 for ED visits and p < 0.001 for hospitalization). In the public system, higher primary care effectiveness attenuated the association between multimorbidity and hospitalization (interaction prevalence ratio [PR] = 0.74, 95% confidence interval [CI] = 0.63–0.87). In contrast, in the private system, higher primary care effectiveness strengthened the associations of multimorbidity with ED visits (interaction PR = 1.40, 95% CI = 1.18–1.66) and hospitalization (interaction PR = 1.75, 95% CI = 1.22–2.50).

**Interpretation:**

Associations between multimorbidity and healthcare use varied according to patient-reported primary care experiences and health system sector.

**Funding:**

The Brazilian National Council for Scientific and Technological Development, the Alzheimer’s Association, and the National Institute on Aging.

**Research in context**

**Evidence before this study**

Multimorbidity affects 30–65% of adults aged ≥50 years in high- and middle-income countries and is consistently associated with greater emergency department (ED) use and hospitalization. Prior studies suggest that primary care may reduce avoidable hospital use, particularly when care is continuous and coordinated. However, most evidence relies on macro-level indicators of primary care (e.g., provider density, coverage, or system-level reforms) from high-income settings rather than patient-reported measures of care effectiveness, which capture patients’ experiences of access, continuity, communication, and coordination. We searched MEDLINE from database inception to May 31, 2026, using the following search terms, without language restrictions: (“multimorbidity” OR “multiple chronic conditions”) AND (“primary care” OR “family practice” OR “general practice” OR “continuity of care” OR “care coordination”) AND (“hospitalization” OR “hospital admission” OR “emergency department” OR “emergency room” OR “acute care use” OR “unplanned care” OR “healthcare utilization”) AND (“older adults” OR “aging” OR “aged”). Few studies have examined whether primary care modifies the association between multimorbidity and acute healthcare use. Evidence from low- and middle-income countries remains limited, and nationally representative studies comparing public and private health systems are lacking.

**Added value of this study**

In a nationally representative sample of Brazilians aged ≥50 years, patient-reported experiences related to primary care modified the association between multimorbidity and ED visits and hospitalizations. In the public system, where multidisciplinary primary care teams are responsible for defined communities and coordinate patients’ care over time, more favorable patient-reported primary care experiences were associated with attenuation of the association between multimorbidity and hospitalization. In contrast, in the private sector, where direct access to specialists and hospitals is more common, more favorable primary care experiences were associated with stronger associations of multimorbidity with ED use and hospitalization.

**Implications of all the available evidence**

Multimorbidity poses a major challenge to health systems worldwide. Patient-reported experiences related to primary care may be associated with lower ED visits and hospitalizations when care is organized to coordinate services longitudinally and integrate care. Improving patient-reported experiences of care alone may be insufficient without structural mechanisms that support continuity, accountability, and coordinated referral pathways in fragmented health systems.

## Introduction

As populations age and the burden of disease shifts toward chronic non-communicable diseases, multimorbidity—defined as the co-occurrence of two or more chronic conditions—has emerged as a defining challenge for health systems worldwide.^1^ In high-income countries, the prevalence of multimorbidity among adults aged ≥50 years ranges from about 30% to 45% in most European countries and 64% in the United States, exceeding 80% among those aged ≥85 years.^2^ Similar patterns are observed in middle-income settings such as Brazil,^3^ where about two-thirds of adults aged ≥50 years live with at least two chronic conditions. Hypertension, osteoarthritis, and diabetes are among the most frequent contributors to multimorbidity, which is consistently linked to greater healthcare use, functional decline, and mortality.^4^

Older adults with multimorbidity require complex care, and fragmentation may contribute to emergency department (ED) visits and hospital admissions.^5^ Mechanisms include poor medication reconciliation, inadequate monitoring, and delayed recognition of clinical deterioration.^4^ For instance, an older adult with hypertension and osteoarthritis may receive anti-inflammatory drugs for pain without appropriate blood pressure monitoring, leading to acute kidney injury and a potentially preventable emergency visit.^6^ Such episodes increase costs and burden health systems.

Primary care, characterized by first-contact access, longitudinal continuity, communication, and coordination, has been proposed as a central strategy to address multimorbidity.^7^ High-quality primary care may reduce avoidable hospital use through proactive management and coordinated referral pathways. However, evidence on whether primary care reduces health service utilization among older adults with multimorbidity remains inconsistent and appears to depend on how care is organized and incentivized within health systems.^8^

Available evidence examining the relationship between primary care and healthcare use often relies on macro-level indicators such as coverage or general practitioner density.^9^ These approaches are valuable for evaluating system-wide capacity but may not capture patients’ experiences of access, continuity, communication, and coordination.^10^ Patient-reported measures provide complementary insight into these domains and can be summarized into composite indices reflecting experiences related to primary care, although such measures may also be influenced by individual characteristics and reporting biases.^11^ Patient-reported metrics may therefore complement system-level indicators in clarifying how primary care relates to healthcare use, particularly in low- and middle-income countries.^12^

Brazil operates a universal public health system (SUS), the exclusive source of care for approximately 75% of older adults.^13^ Within SUS, the Family Health Strategy provides geographically defined, team-based primary care with responsibility for registered populations.^14^ About one-quarter of older adults hold private health insurance, which offers faster access to specialists and hospitals, usually with weaker gatekeeping. Although they remain eligible to use SUS services, their care pathways often differ. This dual structure provides an opportunity to examine how patient-reported experiences related to primary care operate across distinct organizational models.^11^

Using a national representative sample of Brazilians aged ≥50 years,^15^ we aimed to examine whether the association between multimorbidity and healthcare use in the past 12 months (ED visits and hospitalizations) differs according to patient-reported primary care effectiveness and whether this relationship varies across public and private health system sectors.

## Methods

### Study design and population

This cross-sectional analysis used data from the 2019–2021 wave of the Brazilian Longitudinal Study of Aging (ELSI-Brazil), a nationally representative cohort of adults aged ≥50 years.^15^ Participants were recruited from municipalities across all five geographic regions using a multistage, stratified cluster sampling design. Survey weights and sampling procedures were applied to account for differential probabilities of selection and nonresponse, ensuring national representativeness. Detailed methods have been described elsewhere.^15^

The ELSI-Brazil study was approved by the Research Ethics Committee of the Oswaldo Cruz Foundation (CAAE: 34649814.3.0000.5091), and this analysis was approved by the University of São Paulo (CAAE: 74610423.8.0000.0068). All participants provided written informed consent in Portuguese. Participants with missing data on exposure, outcomes, or covariates were excluded from the analytic sample.

### Procedures

#### Multimorbidity

The primary exposure was multimorbidity, defined as the presence of ≥2 of 13 self-reported chronic conditions.^3^ These conditions were selected based on their clinical relevance, high prevalence, and burden among adults aged ≥50 years, following an operational approach consistent with prior studies on multimorbidity.^3^, ^4^ The list includes hypertension, diabetes, stroke, heart failure, myocardial infarction, chronic respiratory diseases (e.g., chronic obstructive pulmonary disease, emphysema, chronic bronchitis), asthma, chronic kidney disease, cancer, Parkinson’s disease, Alzheimer’s disease, depression, and arthritis or rheumatism.

#### Healthcare use

The outcomes comprised two separate measures: ED visits and hospitalizations lasting ≥24 h in the previous 12 months.^16^ Outcome information was obtained through self- or proxy-report during structured interviews using standardized questions. Participants were asked whether they had attended an ED in the previous 12 months and whether they had been admitted to a hospital for 24 h or longer during that period. Those who responded “yes” to hospitalization were further asked to report the number of admissions.

#### Primary care effectiveness index

Primary care effectiveness was defined based on participants’ self-reported experiences with their usual source of healthcare.^11^, ^14^ The index was based on the Inter-American Development Bank International Primary Care Survey and has been previously applied in studies from Latin America and Brazil, including analyses using ELSI-Brazil data.^10^, ^14^ Participants answered the questionnaire considering the healthcare professional or service they most commonly used for healthcare, and responses were anchored to this usual source of care. The index comprises 12 indicators grouped into four domains: first-contact access, continuity, communication, and coordination, which are established attributes of high-quality primary care proposed by Starfield and colleagues.^10^ The domain of first-contact access includes questions about the ease of obtaining a medical appointment, the ability to schedule an appointment within 24 h, and access to appointment information by telephone. Continuity refers to ongoing care with the same physician, along with the physician’s awareness of the participant’s current medications and familiarity with their main health concerns. Communication assesses whether the physician listens attentively, provides clear explanations about treatment, and whether the consultation duration is perceived as adequate. Coordination encompasses whether the physician resolves most health issues, provides guidance on referrals, and facilitates the coordination of specialist care. Consistent with previous studies, Likert-scale responses were dichotomized to identify the presence or absence of primary care problems, facilitating interpretability and comparability across analyses.^10^, ^11^, ^14^ Each item was coded as 1 (“always” or “most of the time”) or 0 (“rarely” or “never”). A total score was obtained by summing all binary items, resulting in an index ranging from 0 to 12, with higher scores indicating more effective primary care experiences.^11^, ^14^

#### Covariates

Covariates were selected according to the Andersen Behavioral Model, which classifies determinants of healthcare use into predisposing, enabling, and need-based factors.^11^ All covariates were based on self-report. Predisposing factors included age (years), sex (female or male), self-identified race/ethnicity categorized as White, Black, Brown (“*Pardo*,” referring to individuals of mixed ancestry), Asian, or Indigenous, years of formal education, and marital status (having a partner vs. not). Enabling factors included region of residence (urban or rural), Brazilian macroregions (North, Northeast, Midwest, Southeast, and South), and residence in an area covered by the Family Health Strategy of the Sistema Único de Saúde (SUS).^17^ We also included the main health system sector (public vs. private) accessed, defined by private health insurance status: individuals with private health insurance were classified as having access to the private health system, and those without private health insurance were classified as exclusively users of the public health system.^18^ Need-based factors included smoking status (never, former, or current), binge drinking (≥5 drinks on one occasion for men or ≥4 for women within the past month), and a count of difficulties or need for assistance in performing six activities of daily living (ADLs), including walking, dressing, bathing, transferring, toileting, and eating, scored from 0 (no difficulty) to 6 (difficulty in all activities).^17^

### Statistical analysis

We reported categorical variables as counts and percentages, and continuous variables as means with standard deviations (SD) or medians with interquartile ranges (IQR), depending on data distribution. Participants with and without multimorbidity were compared using the chi-squared test for categorical variables and the independent-sample t-test or Kruskal–Wallis test for continuous variables, as appropriate. We also reported the prevalence of each chronic condition and multimorbidity across age groups and according to healthcare use, including ED visits and hospitalizations.

For the binary outcomes of any ED visit or hospitalization in the past 12 months, we used Poisson regression with robust standard errors, clustering by the five Brazilian macroregions. We first fitted models including the primary variables of interest (multimorbidity and the primary care effectiveness index) and their interaction. We then fitted fully adjusted models controlling for predisposing, enabling, and need-based covariates. Results were expressed as prevalence ratios (PRs) with 95% confidence intervals (CIs) for multimorbidity, per one-standard deviation (1-SD) increase in the primary care effectiveness index, and for their interaction. Because the structure and access to care differ substantially between the public and private health systems in Brazil,^18^ we also tested a three-way interaction (multimorbidity × primary care effectiveness index × health system sector [public vs. private]) and presented stratified estimates when significant. From the adjusted models, we derived and plotted marginal predicted probabilities for ED visit and hospitalization across the observed range of the primary care effectiveness index, stratified by multimorbidity status and health system sector as appropriate. Fully adjusted models were additionally stratified by sex.

For the count outcome—the number of hospitalizations in the past 12 months—we used negative binomial regression with robust standard errors and clustering by macroregion to estimate rate ratios (RRs) and 95% confidence intervals (CIs). This model was chosen because the variance of the outcome exceeded its mean, making Poisson regression unsuitable. We applied the same covariate adjustments and interaction structure as in the models for binary outcomes. Results were stratified by health system sector when the three-way interaction was significant. From the adjusted models, we derived marginal predicted numbers of hospitalizations per 100 individuals across the observed range of the primary care effectiveness index, by multimorbidity status and health system sector, as appropriate.

Analyses were based on complete cases. We compared included participants with those excluded due to missing data. All analyses accounted for survey weights for the complex design and were conducted in R version 4.2.3 (R Foundation for Statistical Computing, Vienna, Austria) using a two-sided significance level of 0.05.

### Role of the funding source

The funding agencies had no role in the design, conduct, analysis, interpretation, or writing of the study.

## Results

From the 9949 participants in the 2019–2021 wave of ELSI-Brazil, 891 (8.9%) were excluded due to missing data on hospitalization status (n = 106), chronic conditions (n = 195), primary care effectiveness index (n = 270), or covariates (n = 320), resulting in a final sample of 9058 participants (Appendix, p. 2). Compared with included participants, those excluded were less often female and less frequently partnered, while other sociodemographic characteristics were similar (Appendix, p. 3). Participants had a mean age (SD) of 66.3 (9.9) years, 5450 (55.3%) were female sex, and 7577 (84.1%) resided in urban areas (Table 1). The most common chronic conditions were hypertension (n = 4 750, 52.4%), arthritis or rheumatism (n = 1828, 20.2%), and diabetes (n = 1 664, 18.4%) (Fig. 1A). Multimorbidity was identified in 3442 (38.0%) participants, and its prevalence increased progressively with age (Fig. 1B). Participants with multimorbidity were more likely to be older, female, have less education, report a history of smoking but lower levels of binge drinking, experience functional disability, and have access to the private health system (Table 1).

**Table 1:** Participant characteristics according to multimorbidity status.

Variables	Total	Multimorbidity	
		No	Yes
	n = 9058	n = 5616	n = 3442
Age (years), mean (SD)	66.3 (9.9)	65.0 (9.7)	68.3 (10.1)
Female sex	5450 (55.3%)	3127 (50.2%)	2323 (64.5%)
Race/ethnicity			
White	4213 (46.4%)	2635 (47.1%)	1578 (45.3%)
Black	973 (10.9%)	556 (9.8%)	417 (12.8%)
Brown (*“Pardo”*–admixed ancestry)	3812 (42.2%)	2384 (42.7%)	1428 (41.4%)
Other	60 (0.5%)	41 (0.5%)	19 (0.5%)
Education (years), median (IQR)	5 (3–9)	5 (3–11)	5 (2–9)
Having a partner	4853 (61.1%)	3063 (61.8%)	1790 (59.8%)
Urban residence	7577 (84.1%)	4600 (82.2%)	2977 (87.4%)
Smoking status			
Never	5966 (64.7%)	3763 (66.5%)	2203 (61.6%)
Former	1960 (21.7%)	1077 (18.9%)	883 (26.7%)
Current	1132 (13.6%)	776 (14.6%)	356 (11.7%)
Binge drinking	808 (10.3%)	592 (12.0%)	216 (7.5%)
Any ADL disability	941 (9.6%)	362 (5.7%)	579 (16.6%)
Number of chronic conditions, median (IQR)	1 (0–2)	1 (0–1)	2 (2–3)
Hypertension	4750 (52.4%)	1815 (32.3%)	2935 (85.3%)
Arthritis or rheumatism	1828 (20.2%)	419 (7.5%)	1409 (40.9%)
Diabetes	1664 (18.4%)	237 (4.2%)	1427 (41.5%)
Depression	1177 (13.0%)	234 (4.2%)	943 (27.4%)
Heart failure	403 (4.4%)	28 (0.5%)	375 (10.9%)
Stroke	392 (4.3%)	31 (0.6%)	361 (10.5%)
Cancer	392 (4.3%)	76 (1.4%)	316 (9.2%)
Myocardial infarction	386 (4.3%)	35 (0.6%)	351 (10.2%)
Chronic respiratory diseases	315 (3.5%)	43 (0.8%)	272 (7.9%)
Asthma	275 (3.0%)	31 (0.6%)	244 (7.1%)
Chronic kidney disease	202 (2.2%)	17 (0.3%)	185 (5.4%)
Alzheimer’s disease	124 (1.4%)	14 (0.2%)	110 (3.2%)
Parkinson’s disease	80 (0.9%)	11 (0.2%)	69 (2.0%)
Family Health Strategy coverage	5599 (63.0%)	3376 (62.1%)	2223 (64.6%)
Primary care effectiveness index, median (IQR)	9 (7–11)	9 (7–11)	9 (8–11)
Private health system sector	1723 (20.2%)	1000 (18.9%)	723 (22.4%)
Brazilian macroregions			
North	689 (6.7%)	438 (6.9%)	251 (6.4%)
Northeast	2315 (27.3%)	1445 (27.8%)	870 (26.3%)
Southeast	3781 (43.3%)	2351 (42.2%)	1430 (45.2%)
South	1261 (13.7%)	796 (14.3%)	465 (12.6%)
Midwest	1012 (9.1%)	586 (8.8%)	426 (9.5%)

Data are n (%) or mean (SD) or median (IQR). SD = standard deviation; IQR = interquartile range; ADL = activities of daily living (bathing, dressing, toileting, transferring, eating, walking). Multimorbidity was defined as the presence of ≥2 chronic diseases among the following 13 conditions: hypertension, diabetes, heart failure, myocardial infarction, stroke, chronic kidney disease, chronic obstructive pulmonary disease (COPD) or chronic bronchitis/emphysema, asthma, cancer, arthritis or rheumatism, depression, Alzheimer’s disease, and Parkinson’s disease. All estimates account for the complex survey design and sampling weights of ELSI-Brazil.

**Fig. 1:**
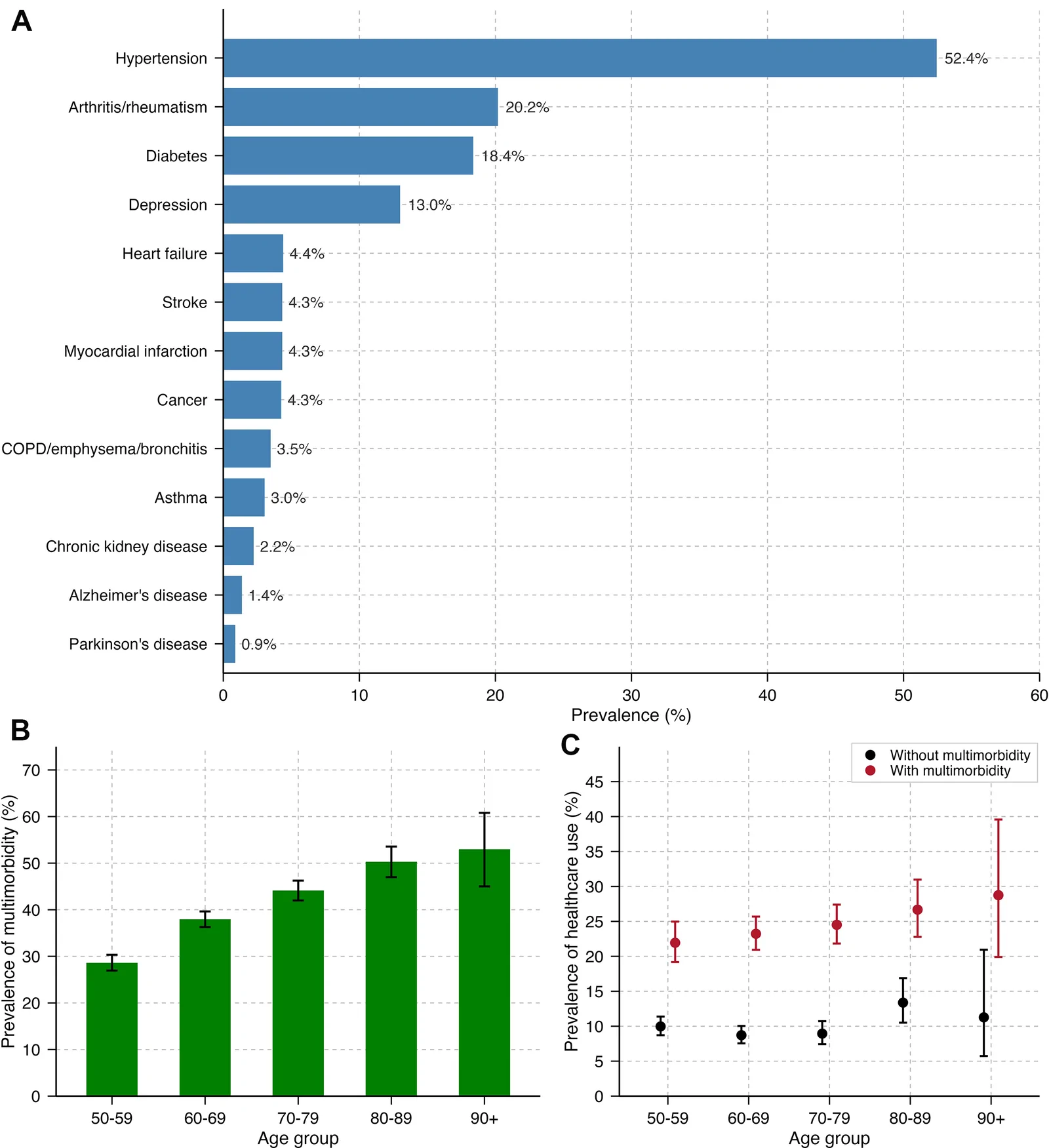
**Multimorbidity patterns and their association with age and healthcare use. Panel A**, the prevalence of chronic conditions included in the multimorbidity index (blue bars); **Panel B**, the prevalence of multimorbidity across age groups (green bars with 95% confidence intervals); **Panel C**, the prevalence of healthcare use, defined as any emergency department visit or hospitalization in the past 12 months, by age group and multimorbidity status, with estimates and their 95% confidence intervals shown in black for individuals without multimorbidity and in red for those with multimorbidity.

The median value of the primary care effectiveness index was 9 (IQR = 7–11), with participants classified as multimorbidity reporting higher scores than those without (9 [IQR = 8–11] vs. 9 [IQR = 7–11], p < 0.001) (Table 1). Participants with multimorbidity reported significantly better continuity, communication, and care coordination with their physicians, while differences in first-contact access were generally small compared with those without multimorbidity (Appendix, p. 4). When stratified by health system sectors, individuals with access to the private system reported higher scores than those using the public system, both among participants with multimorbidity (10 [IQR = 9–12] vs. 9 [IQR = 7–11], p < 0.001) and those without (10 [IQR = 9–12] vs. 9 [IQR = 6–10], p < 0.001) (Appendix, p. 5). Participants with access to the private health system were also older, more educated, more likely to live in urban areas and to identify as White race/ethnicity, and had a higher prevalence of multimorbidity (Appendix, p. 6).

Overall, 1120 (12.5%) of participants reported an ED visit in the past 12 months, 567 (6.0%) had at least one hospitalization, and 153 (1.6%) had two or more hospitalizations (Appendix, p. 7). Healthcare use was consistently higher among individuals with multimorbidity than among those without, across all age groups (Fig. 1C). Regarding health system sectors, the prevalence of ED visits (public = 12.6% [906/7335] vs. private = 12.4% [214/1723]; p = 0.94) and hospitalizations (public = 5.7% [443/7335] vs. private = 7.1% [124/1723]; p = 0.07) in the previous 12 months did not differ substantially. Among participants aged 50–59 years, the prevalence of healthcare use (defined as any ED visit or hospitalization in the past year) was 10.0% (195/1956; 95% CI = 8.7–11.4) in those without multimorbidity and 21.9% (172/784; 95% CI = 19.2–25.0) in those with multimorbidity (p < 0.001). In the oldest group (≥90 years), the corresponding prevalence rates were 11.3% (8/71; 95% CI = 5.7–20.9) and 28.8% (23/80; 95% CI = 19.9–39.6), respectively (p = 0.008).

After adjusting for predisposing, enabling, and need-based factors, participants with multimorbidity had approximately twice the prevalence of ED visits and three times the prevalence of hospitalization compared with those without multimorbidity (Table 2). Higher scores on the primary care effectiveness index were associated with hospitalization overall; however, the interaction between the index and multimorbidity differed by health system sector.

**Table 2:** Interaction between multimorbidity and primary care effectiveness on emergency department visits and hospitalizations in the past 12 months.

	Prevalence ratio (95% confidence interval) p-value	
	Model 1 (Base model)	Model 2 (Fully adjusted)
Emergency department visit		
Whole sample (n = 9058)		
Multimorbidity	2.56 (2.14–3.07) p < 0.001	2.20 (1.90–2.56) p < 0.001
Primary care effectiveness index (per 1-SD increase)	0.98 (0.81–1.20) p = 0.88	1.01 (0.79–1.29) p = 0.94
Interaction between multimorbidity and primary care effectiveness index		
No multimorbidity × primary care effectiveness index	(reference)	(reference)
Multimorbidity × primary care effectiveness index	0.97 (0.89–1.05) p = 0.42	0.94 (0.82–1.07) p = 0.35
Public Health System (n = 7335)		
Multimorbidity	2.52 (2.15–2.95) p < 0.001	2.22 (1.91–2.58) p < 0.001
Primary care effectiveness index (per 1-SD increase)	1.01 (0.79–1.28) p = 0.96	1.00 (0.79–1.28) p = 0.97
Interaction between multimorbidity and primary care effectiveness index		
No multimorbidity × primary care effectiveness index	(reference)	(reference)
Multimorbidity × primary care effectiveness index	0.92 (0.82–1.04) p = 0.17	0.93 (0.82–1.06) p = 0.27
Private Health System (n = 1723)		
Multimorbidity	2.31 (1.98–2.68) p < 0.001	1.89 (1.42–2.51) p < 0.001
Primary care effectiveness index (per 1-SD increase)	0.86 (0.73–1.01) p = 0.06	0.84 (0.73–0.97) p = 0.02
Interaction between multimorbidity and primary care effectiveness index		
No multimorbidity × primary care effectiveness index	(reference)	(reference)
Multimorbidity × primary care effectiveness index	1.29 (0.99–1.68) p = 0.06	1.40 (1.18–1.66) p < 0.001
Hospitalization		
Whole sample (n = 9058)		
Multimorbidity	3.31 (2.93–3.74) p < 0.001	3.00 (2.58–3.49) p < 0.001
Primary care effectiveness index (per 1-SD increase)	1.39 (1.24–1.56) p < 0.001	1.41 (1.18–1.69) p < 0.001
Interaction between multimorbidity and primary care effectiveness index		
No multimorbidity × primary care effectiveness index	(reference)	(reference)
Multimorbidity × primary care effectiveness index	0.82 (0.67–1.02) p = 0.08	0.75 (0.63–0.88) p < 0.001
Public Health System (n = 7335)		
Multimorbidity	3.44 (2.98–3.98) p < 0.001	2.96 (2.56–3.42) p < 0.001
Primary care effectiveness index (per 1-SD increase)	1.43 (1.22–1.67) p < 0.001	1.41 (1.17–1.69) p < 0.001
Interaction between multimorbidity and primary care effectiveness index		
No multimorbidity × primary care effectiveness index	(reference)	(reference)
Multimorbidity × primary care effectiveness index	0.76 (0.64–0.90) p = 0.002	0.74 (0.63–0.87) p < 0.001
Private Health System (n = 1723)		
Multimorbidity	1.80 (1.45–2.24) p < 0.001	1.74 (1.36–2.22) p < 0.001
Primary care effectiveness index (per 1-SD increase)	1.07 (0.74–1.55) p = 0.72	1.13 (0.74–1.71) p = 0.58
Interaction between multimorbidity and primary care effectiveness index		
No multimorbidity × primary care effectiveness index	(reference)	(reference)
Multimorbidity × primary care effectiveness index	1.91 (1.44–2.53) p < 0.001	1.75 (1.22–2.50) p = 0.002

SD = standard deviation (1-SD increase in the primary care effectiveness index corresponded to 3.1 points in the overall sample).

Estimates were computed using Poisson regression models with robust standard errors and adjustment for clustering by the five Brazilian macroregions. Multimorbidity was defined as the presence of ≥2 of 13 chronic conditions, with participants with <2 conditions serving as the reference group.

Model 1 is unadjusted and includes the interaction between multimorbidity and standardized primary care effectiveness.

Model 2 is fully adjusted for age, sex, race/ethnicity, education, marital status, place of residence (urban vs. rural), smoking, binge drinking, Family Health Strategy coverage, and functional disability, defined as the number of difficulties in six self-care activities (bathing, dressing, eating, toileting, transferring, and walking).

Significant three-way interactions (multimorbidity × primary care effectiveness × health system sector) were found for emergency department visit (p = 0.04) and hospitalization (p < 0.001); therefore, results are also presented stratified by access to different health system sectors in Brazil (public vs. private).

A significant three-way interaction was observed between multimorbidity, the primary care effectiveness index, and health system sector for ED visits and hospitalizations, with interaction p-values of 0.04 and < 0.001, respectively. In the public system, higher scores on the primary care effectiveness index were associated with attenuation of the association between multimorbidity and hospitalization, with an interaction PR of 0.74 (95% CI = 0.63–0.87). By contrast, in the private system, higher scores on the primary care effectiveness index were associated with stronger associations between multimorbidity and healthcare use, with interaction PRs of 1.40 (95% CI = 1.18–1.66) for ED visits and 1.75 (95% CI = 1.22–2.50) for hospitalizations (Table 2). In the public system, as scores on the primary care effectiveness index increased, the difference in predicted probability of hospitalization between participants with and without multimorbidity narrowed; although a similar pattern was observed for ED visits, the interaction was not statistically significant (Fig. 2). In contrast, in the private system, the predicted probabilities of ED visits and hospitalization diverged, with a progressive increase in events among those with multimorbidity and a decrease or stabilization among those without multimorbidity. Sex-stratified analyses showed patterns consistent with the primary findings, without evidence of major sex-related differences (Appendix, p. 8).

**Fig. 2:**
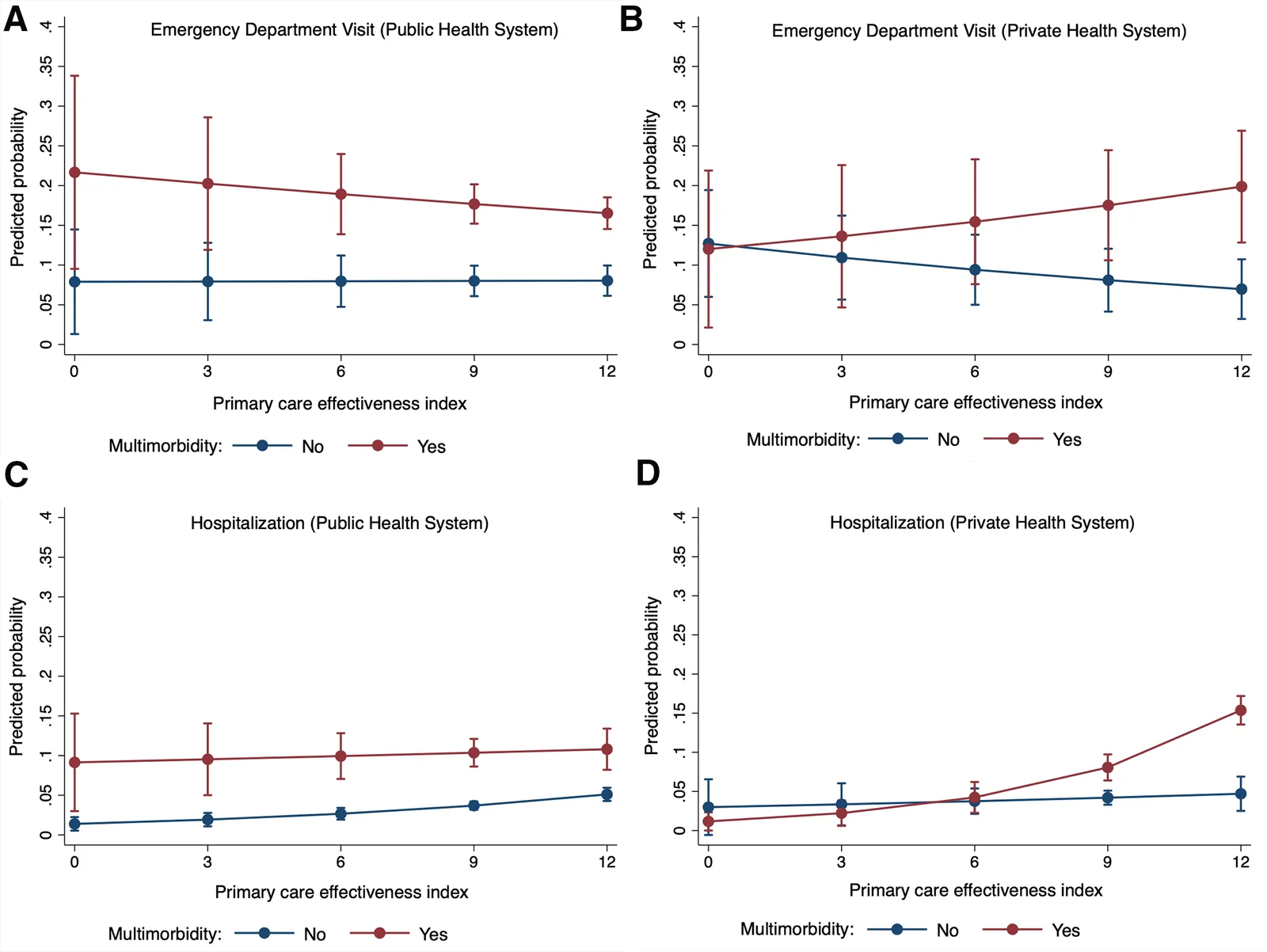
**Predicted healthcare use according to multimorbidity status, primary care effectiveness index score, and health system sector in Brazil**. (A) Emergency department visits in the public health system; (B) Emergency department visits in the private health system; (C) Hospitalizations in the public health system; and (D) Hospitalizations in the private health system.

Analyses that considered the number of hospitalizations in the past 12 months as the outcome showed similar findings. In the overall sample, multimorbidity was associated with a higher hospitalization rate in the past 12 months (adjusted RR = 3.81, 95% CI = 2.88–5.04). Higher scores on the primary care effectiveness index were also associated with a higher rate of hospitalizations (adjusted RR per 1-SD increase in the index [3.1 points] = 1.54, 95% CI = 1.32–1.79). However, a significant three-way interaction was also observed between multimorbidity, the primary care effectiveness index, and health system sector for the number of hospitalizations, with an interaction p-value of 0.003 (Appendix, p. 9). In the public system, higher scores on the primary care effectiveness index were associated with lower hospitalization rates among participants with multimorbidity (interaction RR = 0.64, 95% CI = 0.48–0.85). By contrast, in the private system, higher scores on the primary care effectiveness index were associated with higher hospitalization rates (interaction RR = 1.35, 95% CI = 1.07–1.70). Predicted mean hospitalizations per 100 individuals across primary care effectiveness index scores demonstrated divergent patterns among participants with multimorbidity: hospitalization rates declined as index scores increased in the public sector but rose with increasing scores in the private sector (Fig. 3).

**Fig. 3:**
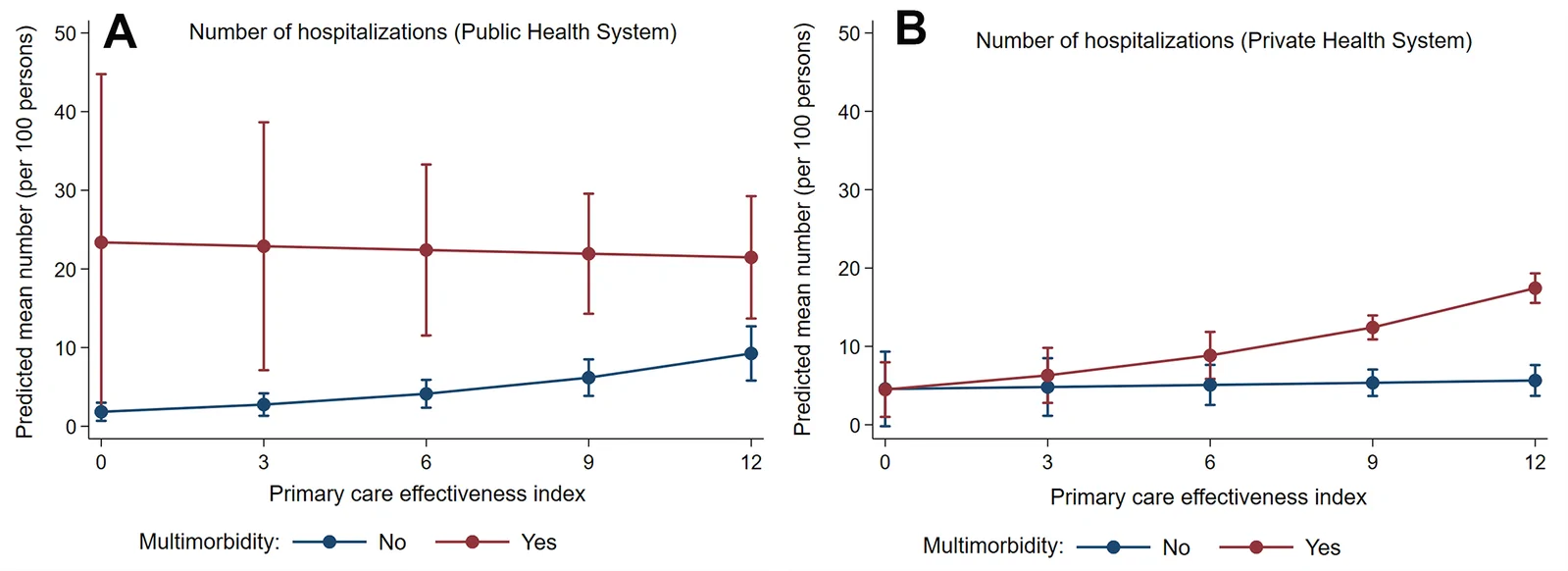
**Predicted mean number of hospitalizations in the past 12 months by primary care effectiveness index score and health system sector in Brazil.Panel A shows hospitalizations in the public health system, and panel B shows hospitalizations in the private health system.** Estimates were computed from negative binomial regression models with robust standard errors and adjustment for clustering by the five Brazilian macroregions. Models also incorporated age, sex, race/ethnicity, education, marital status, place of residence (urban vs. rural), smoking, binge drinking, Family Health Strategy coverage, and functional disability. Estimates represent the predicted mean number of hospitalizations per 100 persons in the past year, with 95% confidence intervals.

## Discussion

In this nationally representative study, multimorbidity was common and strongly associated with ED visits and hospitalizations. However, associations with the patient-reported primary care effectiveness index differed across Brazil’s public and private sectors. In the public system, higher scores on the primary care effectiveness index attenuated the association between multimorbidity and hospitalization. By contrast, in the private sector, higher scores on the primary care effectiveness index were associated with stronger associations of multimorbidity with ED visits and hospitalizations. These contrasting patterns persisted when modelling the number of hospitalizations.

Our results add to prior evidence showing that multimorbidity is common and strongly associated with healthcare use.^16^ A US population-based study found that nearly one in three adults aged ≥50 years with ≥2 chronic conditions reported at least one ED visit in the past 12 months.^19^ In addition, a large systematic review and meta-analysis of 33 observational studies in adults aged ≥60 years across diverse settings reported that multimorbidity was associated with higher odds of hospitalization and readmission.^4^ While this higher–risk profile likely reflects greater clinical complexity, treatment burden, and susceptibility to acute decompensation among people living with multiple chronic conditions, prior conceptual work suggests that fragmentation and limited coordination in ambulatory care may also contribute to increased hospital use.^20^ Consistent with this framework, our findings suggest that associations between multimorbidity and healthcare use may vary according to patient-reported experiences with a usual source of healthcare.

In interpreting these findings, patient-reported primary care effectiveness may operate differently depending on how care is organized and incentivized. The primary care effectiveness index used here comprises 12 items that capture participants’ experiences with access, continuity, communication, and coordination with their usual source of healthcare.^11^ Higher scores, therefore, indicate more favorable reported experiences, but they do not necessarily imply gatekeeping, integrated information systems, or constraints on specialist referral and hospital access.^14^ Because responses were anchored to participants’ usual source of healthcare, the services being evaluated may not represent equivalent models of care across health system sectors. In settings where primary care coexists with direct access to specialists and hospitals, reported experiences may not always refer exclusively to primary care physicians, and higher scores on the primary care effectiveness index may reflect responsiveness and convenience that accelerate referrals, diagnostic cascades, and admissions among high-need patients, rather than preventing care use.^21^ By contrast, in settings where primary care is structurally positioned to coordinate care and reduce potentially avoidable use of acute services, higher effectiveness may be associated with fewer ED visits and hospital admissions.^22^ Comparative studies suggest that integrated primary care systems are associated with fewer potentially avoidable secondary-care visits than fragmented systems.^23^

Brazil’s dual health system offered a unique opportunity to examine how organizational context influences the implications of patient-reported primary care effectiveness. In the public sector, the Family Health Strategy (FHS) provides geographically defined, team-based primary care with accountability for registered populations, supporting continuity, coordination, and earlier recognition of clinical deterioration.^24^ Evidence indicates that FHS expansion has reduced ambulatory care sensitive conditions and improved chronic disease control, suggesting meaningful capacity to substitute for emergency and hospital care.^25^ This organizational design may explain why the association between multimorbidity and hospitalization was attenuated as primary care effectiveness index scores increased in the public system.^22^ By contrast, Brazil’s private sector typically features weaker gatekeeping and more direct access to specialists, diagnostic exams, and hospitals, often positioning primary care as a complement to specialized services rather than the locus of care coordination.^26^ In fee-for-service environments, incentives may further favor referral and intervention over conservative longitudinal management. Prior work shows that older adults with private health plans use more services and incur higher expenditures than those relying exclusively on the public system.^27^ In our study, higher scores on the primary care effectiveness index in the private sector may have captured convenience and rapid referral pathways that increase ED visits and admissions, rather than preventing them.^28^

As multimorbidity becomes more prevalent with population aging, strengthening primary care remains a central strategy to improve health system performance.^9^, ^12^ Our findings add nuance by indicating that patient-reported primary care effectiveness may attenuate the association between multimorbidity and hospitalization when primary care is structurally positioned to coordinate services. In the public sector, findings support continued investment in longitudinal, team-based care and structured coordination.^29^ In the private sector, aligning incentives toward coordination, such as clearer referral pathways and interoperable records, may be necessary.^29^ More broadly, these results are relevant for other mixed or segmented systems, suggesting that improving primary care experiences alone may be insufficient without complementary organizational mechanisms that support continuity and navigation across care pathways.

Some limitations should be considered. The cross-sectional design precludes causal inference and raises the possibility of reverse causation, whereby recent ED visits or hospitalizations may influence contact with primary care and reported experiences. Data were collected between 2019 and 2021, overlapping with the COVID-19 pandemic, which may have influenced healthcare use and reported primary care experiences. Measures of multimorbidity, healthcare use, and primary care effectiveness were self-reported, which may introduce recall and reporting biases. Although the patient-reported primary care effectiveness index was based on established attributes of primary care proposed by Starfield and colleagues and has been previously applied in multinational and ELSI-Brazil studies,^10^, ^11^, ^14^ it does not represent a comprehensive psychometric instrument such as the Primary Care Assessment Tool (PCAT). The use of this measure was driven by data availability within ELSI-Brazil and comparability with previous population-based studies using the same methodology. Patient-reported primary care experiences may reflect dimensions of care quality that are only partially correlated with objective quality indicators derived from routine clinical data.^30^ In addition, because responses were anchored to participants’ usual source of healthcare, the services evaluated may not represent equivalent care settings across health system sectors. Furthermore, dichotomization of Likert-scale responses may have reduced sensitivity to detect gradations in these experiences. Residual confounding also cannot be excluded, including unmeasured variation in clinical severity and disease control, care-seeking preferences, plan generosity, provider practice styles, and socioeconomic factors not fully captured by educational attainment. The multimorbidity definition based on ≥2 chronic conditions may not fully capture disease burden, functional impairment, or psychiatric comorbidities beyond depression. Moreover, prevalence estimates of multimorbidity vary with the number and types of chronic conditions included, which may limit comparisons with previous work.^4^ Such confounding may be particularly relevant in the private sector, where individuals with greater need may have more frequent contact with their usual source of healthcare, rate it more favorably, and still experience higher utilization due to underlying risk.^28^ Finally, the health system sector was classified based on private health insurance status and may not fully capture actual care pathways, as some individuals may use public and private services. Nevertheless, this misclassification would most likely have attenuated, rather than exaggerated, contrasts between sectors.

Some important strengths should also be noted. This study used a large, nationally representative sample of Brazilians aged ≥50 years. The analyses were grounded in Andersen’s behavioral model, with adjustment for predisposing, enabling, and need-related factors, which helps to reduce confounding by key sociodemographic and clinical characteristics.^11^ Notably, we found consistent evidence of effect modification across outcomes, including a three-way interaction between multimorbidity, primary care effectiveness, and the health system sector. This finding illustrates that the same patient-reported measure of experiences related to primary care can have different implications depending on the structure of the underlying care model and health system context.^20^

In this nationally representative study, multimorbidity was common and associated with higher ED visits and hospitalizations among adults aged ≥50 years. These associations differed according to patient-reported primary care experiences and health system sector. In the public system, more favorable patient-reported primary care experiences were associated with attenuation of the association between multimorbidity and hospitalization; in the private sector, more favorable primary care experiences were associated with a stronger association between multimorbidity and healthcare use. These findings support the importance of healthcare system context when interpreting associations between patient-reported primary care experiences and healthcare utilization.

## Contributors

GMO: conceptualization, data curation, investigation, formal analysis, methodology, visualization, writing − original draft, and writing − review & editing.

BPN: conceptualization, investigation, methodology, and writing − review & editing.

KL: funding acquisition, conceptualization, methodology, and writing − review & editing. JLT, JM, BSM, TJAS, VADG, SQFF, TFC, SRRB: conceptualization, methodology, and writing − review & editing.

MFLC: funding acquisition, conceptualization, methodology, and writing − review & editing. EF: conceptualization, investigation, methodology, vizualisation, and writing − review & editing.

MJRA: funding acquisition, conceptualization, data curation, investigation, project administration, supervision, formal analysis, methodology, visualization, writing − original draft, and writing − review & editing.

GMO and MJRA accessed and verified the underlying data. All authors had full access to the data and had final responsibility for the decision to submit for publication.

## Data sharing statement

The Brazilian Longitudinal Study of Aging (ELSI-Brazil) data are freely available to researchers upon registration with the ELSI-Brazil project at the following address https://elsi.cpqrr.fiocruz.br/en/register/.

## Declaration of interests

The authors have no competing interests.
