# Supplementary material for: Primary care effectiveness, multimorbidity, and hospital use in public and private health-care systems in Brazil: a cross-sectional analysis of data from the 2019–2021 Brazilian longitudinal study of aging
Source: Lancet Reg Health Am. 2026 Oct 17;66:101680. doi: 10.1016/j.lana.2026.101680 (PMC13696340; doi:10.1016/j.lana.2026.101680)
Supplement: Supplementary file 1 [file mmc1.pdf]

Primary care effectiveness, multimorbidity, and hospital use in public and private health-care systems in Brazil: a cross-sectional analysis of data from the 2019–21 Brazilian Longitudinal Study of Aging

This supplementary appendix for online-only contains:

Figures

Flowchart of the study participants (p. 2)

Distribution of primary care effectiveness index by multimorbidity status and type of health system sector (public *vs* private) in Brazil (n=9 058) (p. 5)

Tables

Characteristics of the individuals according to their participation status (p. 3)

Primary care experiences according to multimorbidity status among older adults (p. 4)

Participant characteristics by health system sectors (private vs public) (p. 6)

Healthcare use in the past 12 months according to multimorbidity status (p. 7)

Interaction between multimorbidity and the primary care effectiveness index on emergency department visits and hospitalizations across health system sectors, stratified by sex (p. 8)

Interaction between multimorbidity and the primary care effectiveness index on the rate of hospitalizations in the past 12 months (p. 9)

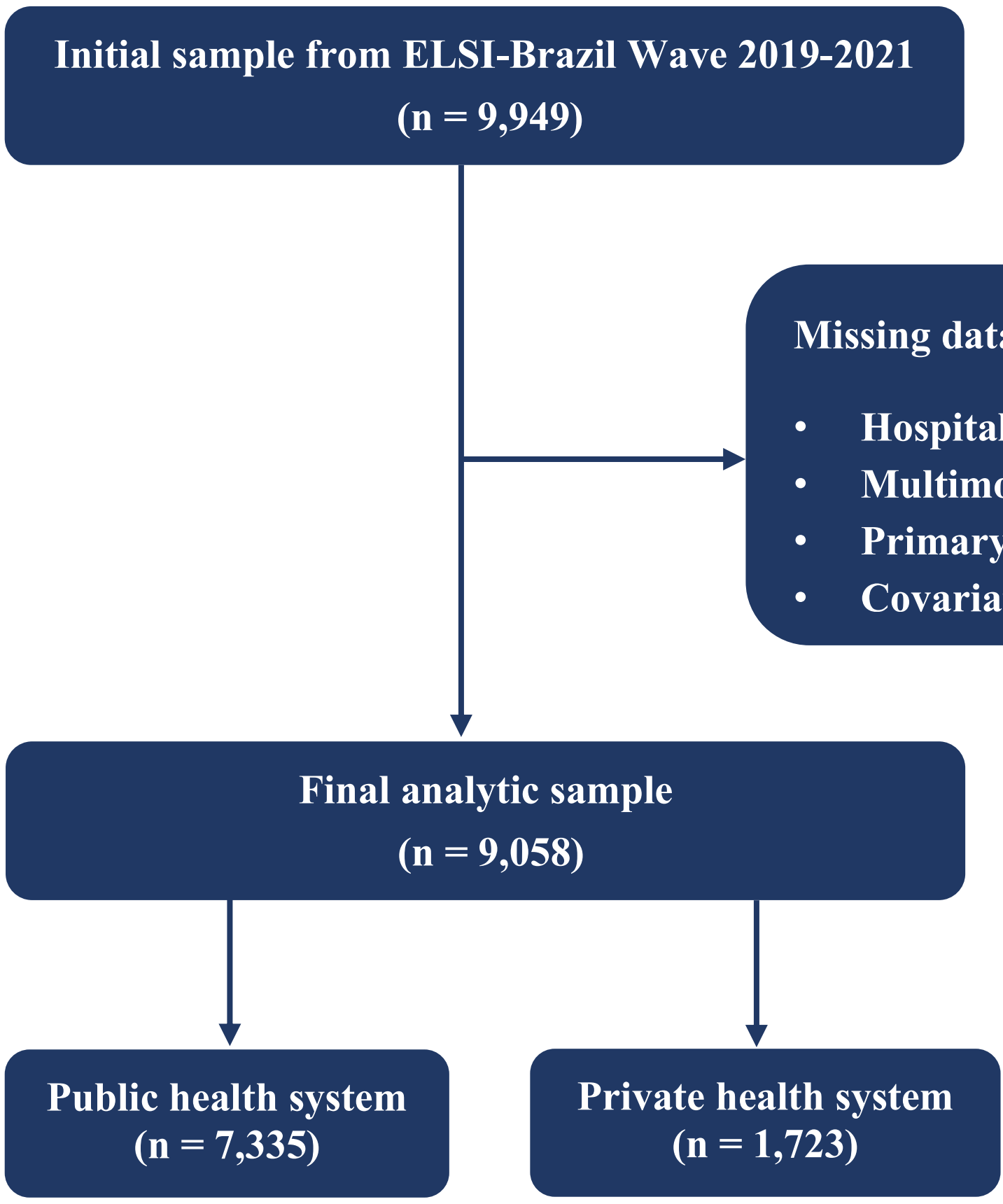

Flowchart of the study participants

ELSI-Brazil = Brazilian Longitudinal Study of Aging.

Characteristics of the individuals according to their participation status

Variables	Included (n=9 058)	Excluded due to missing data (n=891)	p-value
Age group (years)			0.08
50–64	62·5%	61·2%	
65–79	30·2%	29·2%	
≥ 80	7·3%	9·6%	
Female sex	55·3%	44·4%	<0.001
White race/ethnicity	46·4%	46·1%	0.93
Education <8 years	63·3%	64·9%	0.66
Having a partner	61·1%	55·2%	0.01
Urban residence	84·1%	88·1%	0.17
Private health system	20·2%	14·2%	0.05

Primary care experiences according to multimorbidity status among older adults

Domains	Questions on primary care experiences	Total	Multimorbidity		p-value
			No	Yes	
		(n=9,058)	(n=5,616)	(n=3,442)	
**First-contact access**	Do you find it easy to obtain a medical appointment?	64.4%	63.3%	66.1%	0.006
	Are you able to obtain a medical appointment within 24 hours when you are ill?	50.6%	50.3%	51.2%	0.39
	Are you able to obtain health-related information by telephone when you need care, either through the health service or directly from a physician?	40.4%	40.8%	39.6%	0.27
**Continuity of care**	Have you seen the same physician who attended you on previous visits?	65.7%	61.5%	72.3%	<0·001
	Does the physician who attends you know about all the medications you are currently taking?	83.0%	79.7%	88.4%	<0·001
	Does the physician who attends you know your main health problems (i.e., has a medical record or chart with this information)?	81.8%	78.5%	87.2%	<0·001
**Communication**	Does the physician who attends you listen carefully to your concerns?	85.8%	83.5%	89.6%	<0·001
	Does the physician who attends to you explain things about your health or treatment in a way that you can understand?	87.1%	85.1%	90.4%	<0·001
	Do you feel your medical consultation lasted the necessary amount of time?	82.0%	80.2%	84.9%	<0·001
**Coordination**	Is the physician who attends you able to resolve most of your health problems?	85.9%	84.5%	88.1%	<0·001
	How often does the general practitioner talk to you about the results of your specialist consultation?	66.9%	65.6%	69.1%	<0·001
	How often does the provider who attends to you (a general practitioner or another health professional) help you schedule tests or a specialist appointment?	61.6%	60.2%	63.7%	0.005

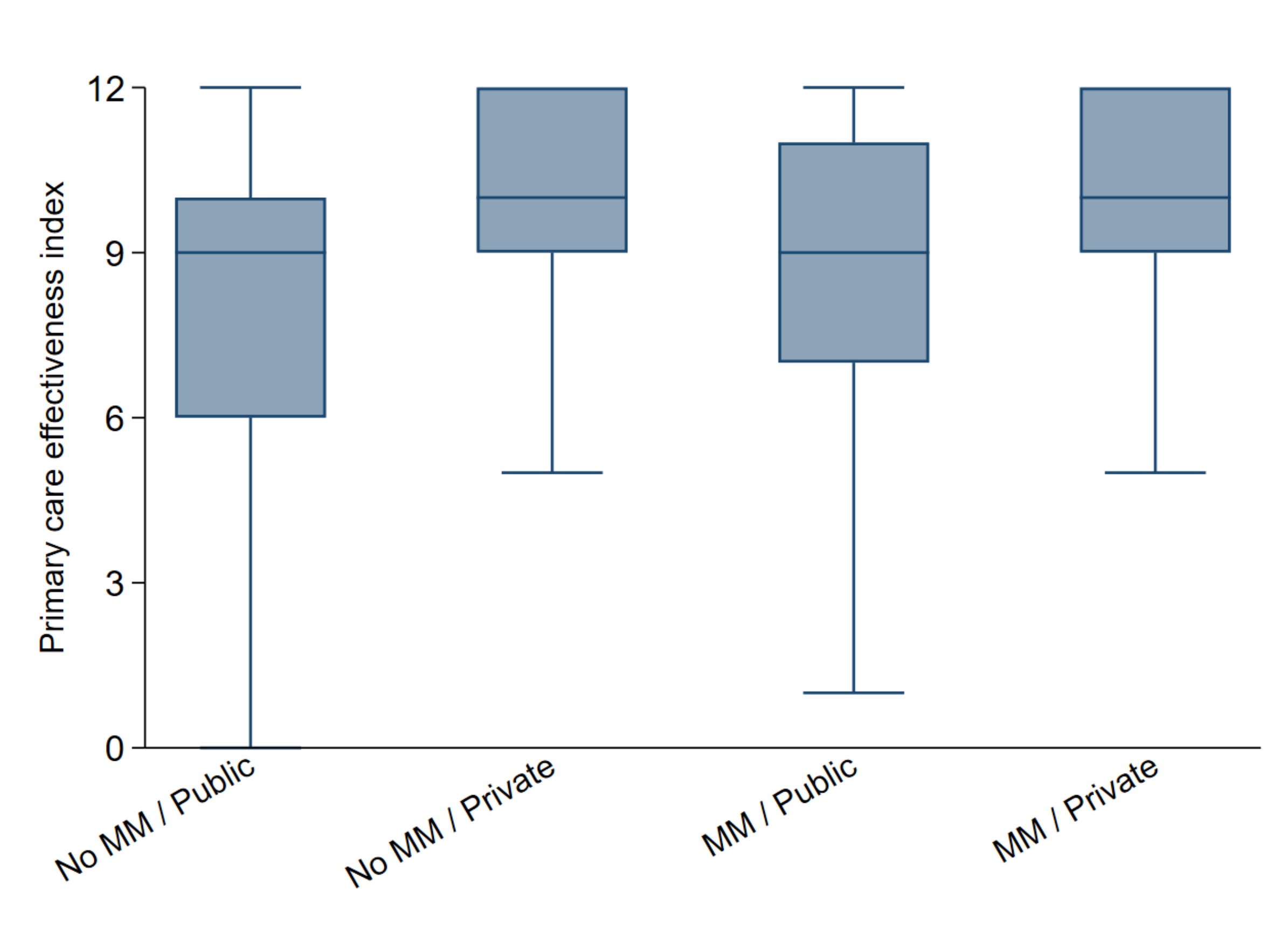

Distribution of primary care effectiveness index by multimorbidity status and type of health system sector (public *vs* private) in Brazil (n=9 058)

MM = Multimorbidity (defined as having two or more chronic diseases among a list of 13 chronic conditions). Public and Private refer to the type of health system reported by participants as their usual source of care. Among participants with and without multimorbidity, those with access to the private health system had higher median scores on the primary care effectiveness index than those using the public system ($p<0{\cdot}001$ for both comparisons).

Participant characteristics by health system sectors (private *vs* public)

Variables	Total<br>n = 9 058	Healthcare Sector<br>Private<br>n =1 723	 Public<br>n = 7 335
Age (years), mean (SD)	66·3 (9·9)	67·5 (10·2)	66·0 (9·9)
Female sex	5 450 (55·3%)	1 068 (55·1%)	4 382 (55·4%)
Race/ethnicity			
White	4 213 (46·4%)	1 018 (57·3%)	3 195 (43·7%)
Black	973 (10·9%)	133 (7·7%)	840 (11·7%)
Brown (*"Pardo"*–admixed ancestry)	3 812 (42·2%)	563 (34·3%)	3 249 (44·2%)
Other	60 (0·5%)	9 (0·6%)	51 (0·4%)
Education (years), median (IQR)	5 (3–9)	9 (5–12)	5 (2–9)
Having a partner	4 853 (61·1%)	994 (67·9%)	3 859 (59·4%)
Urban residence	7 577 (84·1%)	1621 (95·1%)	5 956 (81·3%)
Smoking status			
Never	5 966 (64·7%)	1 195 (68·8%)	4 771 (63·7%)
Former	1 960 (21·7%)	389 (23·7%)	1571 (21·2%)
Current	1 132 (13·6%)	139 (7·5%)	993 (15·1%)
Binge drinking	808 (10·3%)	156 (9·9%)	652 (10·4%)
Any ADL disability	941 (9·6%)	147 (8·0%)	794 (10·0%)
Number of chronic conditions, median (IQR)	1 (0–2)	1 (0–2)	1 (0–2)
Multimorbidity (≥2 chronic diseases)	3442 (38·0%)	723 (42·0%)	2 719 (37·1%)
Hypertension	4 750 (52·4%)	930 (54·0%)	3 820 (52·1%)
Arthritis or rheumatism	1 828 (20·2%)	383 (22·2%)	1 445 (19·7%)
Diabetes	1 664 (18·4%)	341 (19·8%)	1 323 (18·0%)
Depression	1 177 (13·0%)	243 (14·1%)	934 (12·7%)
Heart failure	403 (4·4%)	92 (5·3%)	311 (4·2%)
Stroke	392 (4·3%)	61 (3·5%)	331 (4·5%)
Cancer	392 (4·3%)	116 (6·7%)	276 (3·8%)
Myocardial infarction	386 (4·3%)	88 (5·1%)	298 (4·1%)
Chronic respiratory diseases	315 (3·5%)	61 (3·5%)	254 (3·5%)
Asthma	275 (3·0%)	63 (3·7%)	212 (2·9%)
Chronic kidney disease	202 (2·2%)	51 (3·0%)	151 (2·1%)
Alzheimer's disease	124 (1·4%)	24 (1·4%)	100 (1·4%)
Parkinson's disease	80 (0·9%)	18 (1·0%)	62 (0·8%)
Family Health Strategy coverage	5 599 (63·0%)	973 (55·2%)	4 626 (65·0%)
Primary care effectiveness index, median (IQR)	9 (7–11)	10 (9–12)	9 (7–11)
Brazilian macroregions			
North	689 (6·7%)	74 (5·8%)	615 (6·9%)
Northeast	2 315 (27·3%)	234 (13·5%)	2 081 (30·8%)
Southeast	3 781 (43·3%)	994 (59·2%)	2 787 (39·2%)
South	1 261 (13·7%)	230 (12·4%)	1 031 (14·0%)
Midwest	1 012 (9·1%)	191 (9·1%)	821 (9·1%)

Data are n (%) or mean (SD) or median (IQR). SD = standard deviation; IQR = interquartile range; ADL = activities of daily living (bathing, dressing, toileting, transferring, eating, walking).
All estimates account for the complex survey design and sampling weights of ELSI-Brazil.

Healthcare use in the past 12 months according to multimorbidity status

Healthcare use, n (%)	Total (n=9,058)	No multimorbidity (n=5,616)	Multimorbidity (n=3,442)	p-value
Emergency department visit	1,120 (12·5%)	439 (8·3%)	681 (20·0%)	<0·001
≥1 hospitalization	567 (6·0%)	188 (3·4%)	379 (10·5%)	<0·001
≥2 hospitalizations	153 (1·6%)	36 (0·6%)	117 (3·5%)	<0·001
Number of hospitalizations				<0·001
0	8,491 (94·0%)	5,428 (96·6%)	3,063 (89·5%)	
1	414 (4·3%)	152 (2·9%)	262 (6·9%)	
2	94 (1·0%)	24 (0·4%)	70 (2·1%)	
3	31 (0·3%)	6 (0·1%)	25 (0·8%)	
4	13 (0·2%)	3 (0·0%)	10 (0·3%)	
≥ 5	15 (0·2%)	3 (0·0%)	12 (0·4%)	

Data are unweighted counts and survey-weighted percentages. Percentages account for the complex survey design and sampling weights.

Interaction between multimorbidity and the primary care effectiveness index on emergency department visits and hospitalizations across health system sectors, stratified by sex

	Prevalence ratio (95% confidence interval) p-value	
	**Female sex (n=5 450)**	**Male sex (n = 3 608)**
**Emergency Department Visit**		
Whole sample		
Multimorbidity	2·24 (1·91–2·63) p<0·001	2·18 (1·69–2·80) p<0·001
Primary care effectiveness index (per 1-SD increase)	0·96 (0·78–1·17) p=0·67	1·07 (0·79–1·46) p=0·66
Interaction between multimorbidity and primary care effectiveness index		
No multimorbidity × primary care effectiveness index	(reference)	(reference)
Multimorbidity × primary care effectiveness index	0·98 (0·86–1·12) p=0·77	0·88 (0·63–1·22) p=0·44
Public Health System		
Multimorbidity	2·23 (1·95–2·55) p<0·001	2·18 (1·68–2·84) p<0·001
Primary care effectiveness index (per 1-SD increase)	0·95 (0·77–1·16) p=0·60	1·07 (0·79–1·45) p=0·66
Interaction between multimorbidity and primary care effectiveness index		
No multimorbidity × primary care effectiveness index	(reference)	(reference)
Multimorbidity × primary care effectiveness index	0·98 (0·87–1·11) p=0·76	0·88 (0·63–1·21) p=0·43
Private Health System		
Multimorbidity	1·76 (1·39–2·24) p<0·001	2·47 (1·41–4·34) p=0·002
Primary care effectiveness index (per 1-SD increase)	0·86 (0·73–1·02) p=0·07	0·77 (0·40–1·51) p=0·45
Interaction between multimorbidity and primary care effectiveness index		
No multimorbidity × primary care effectiveness index	(reference)	(reference)
Multimorbidity × primary care effectiveness index	1·39 (1·14–1·69) p=0·001	1·56 (0·83–2·91) p=0.17
**Hospitalization**		
Whole sample		
Multimorbidity	3·06 (2·18–4·29) p<0·001	2·89 (2·13–3·94) p<0·001
Primary care effectiveness index (per 1-SD increase)	1·34 (1·04–1·72) p=0·02	1·50 (1·27–1·77) p<0·001
Interaction between multimorbidity and primary care effectiveness index		
No multimorbidity × primary care effectiveness index	(reference)	(reference)
Multimorbidity × primary care effectiveness index	0·74 (0·65–0·84) p<0·001	0·80 (0·59–1·08) p=0·15
Public Health System		
Multimorbidity	3·02 (2·26–4·04) p<0·001	2·88 (2·08–4·00) p<0·001
Primary care effectiveness index (per 1-SD increase)	1·33 (1·03–1·71) p=0·03	1·51 (1·26–1·80) p<0·001
Interaction between multimorbidity and primary care effectiveness index		
No multimorbidity × primary care effectiveness index	(reference)	(reference)
Multimorbidity × primary care effectiveness index	0·74 (0·65–0·83) p<0·001	0·79 (0·58–1·07) p=0·13
Private Health System		
Multimorbidity	1·87 (0·91–3·85) p=0·09	1·79 (0·80–4·02) p=0·16
Primary care effectiveness index (per 1-SD increase)	1·33 (0·50–3·51) p=0·56	0·99 (0·49–2·02) p=0·99
Interaction between multimorbidity and primary care effectiveness index		
No multimorbidity × primary care effectiveness index	(reference)	(reference)
Multimorbidity × primary care effectiveness index	1·75 (0·64–4·79) p=0.28	1·42 (0·40–5·07) p=0·59

SD = standard deviation (1-SD increase in the primary care effectiveness index corresponded to 3·1 points among females and 3·2 among males).

Estimates were computed using Poisson regression models with robust standard errors and adjustment for clustering by the five Brazilian macroregions. Multimorbidity was defined as the presence of ≥2 of 13 chronic conditions, with participants with <2 conditions serving as the reference group. Models were adjusted for age, race/ethnicity, education, marital status, urban versus rural residence, smoking, binge drinking, Family Health Strategy coverage, and functional disability. Among public system users, 4 382/7 335 (59·7%) were female and 2 953/7 335 (40·3%) were male. Among private system users, 1 068/1 723 (62·0%) were female and 655/1 723 (38·0%) were male. For females, p-values for the three-way interactions (multimorbidity × primary care effectiveness index × health system sector) were 0·13 for ED visits and 0·07 for hospitalizations; for males, corresponding p-values were 0·10 and 0·25.

Interaction between multimorbidity and the primary care effectiveness index on the rate of hospitalizations in the past 12 months

	Rate ratio (95% confidence interval) p-value	
	**Model 1 (Base model)**	**Model 2 (Fully adjusted)**
Whole sample (n=9,058)		
Multimorbidity		
No	(reference)	(reference)
Yes	4·21 (3·44–5·14) p<0·001	3·81 (2·88–5·04) p<0·001
Primary care effectiveness index (per 1-SD increase)	1·47 (1·30–1·65) p<0·001	1·54 (1·32–1·79) p<0·001
Interaction between multimorbidity and primary care effectiveness		
No multimorbidity × primary care effectiveness index	(reference)	(reference)
Multimorbidity × primary care effectiveness index	0·66 (0·47–0·92) p=0·02	0·64 (0·47–0·87) p=0·004
Public Health System (n=7,335)		
Multimorbidity		
No	(reference)	(reference)
Yes	4·34 (3·50–5·37) p<0·001	3·85 (2·91–5·08) p<0·001
Primary care effectiveness index (per 1-SD increase)	1·55 (1·30–1·85) p<0·001	1·53 (1·32–1·78) p<0·001
Interaction between multimorbidity and primary care effectiveness		
No multimorbidity × primary care effectiveness index	(reference)	(reference)
Multimorbidity × primary care effectiveness index	0·61 (0·43–0·86) p=0·005	0·64 (0·48–0·85) p=0·002
Private Health System (n=1,723)		
Multimorbidity		
No	(reference)	(reference)
Yes	2·39 (1·44–3·98) p=0·001	2·20 (1·51–3·19) p<0·001
Primary care effectiveness index (per 1-SD increase)	1·02 (0·74–1·40) p=0·92	1·06 (0·77–1·44) p=0·72
Interaction between multimorbidity and primary care effectiveness		
No multimorbidity × primary care effectiveness index	(reference)	(reference)
Multimorbidity × primary care effectiveness index	1·50 (1·15–1·96) p=0·003	1·35 (1·07–1·70) p=0·01

Estimates were computed from negative binomial regression models with robust standard errors and adjustment for clustering by the five Brazilian macroregions.

Model 1 is unadjusted and includes the interaction between multimorbidity and the standardized primary care effectiveness index.

Model 2 is fully adjusted for age, sex, race/ethnicity, education, marital status, place of residence (urban vs· rural), smoking, binge drinking, Family Health Strategy coverage, and functional disability, defined as the number of difficulties in six self-care activities (bathing, dressing, eating, toileting, transferring, and walking).

Significant three-way interactions (multimorbidity × primary care effectiveness index × health system sector) were found for the number of hospitalizations (p=0·003); therefore, results are also presented stratified by health system sector (public *vs* private).
